# Chitosan Prevents Gentamicin-Induced Nephrotoxicity via a Carbonyl Stress-Dependent Pathway

**DOI:** 10.1155/2015/675714

**Published:** 2015-04-12

**Authors:** Chu-Kung Chou, Yi-Chieh Li, Shih-Ming Chen, Yi-Min Shih, Jen-Ai Lee

**Affiliations:** ^1^Department of Internal Medicine, Chia-Yi Christian Hospital, 539 Jhongsiao Road, Chiayi City 60002, Taiwan; ^2^Department of Internal Medicine, National Taiwan University Hospital, No. 7, Chung-Shan South Road, Taipei City 10002, Taiwan; ^3^School of Pharmacy, College of Pharmacy, Taipei Medical University, 250 Wuxing Street, Taipei City 11031, Taiwan

## Abstract

Aminoglycosides are widely used to treat infections; however, their applications are limited by nephrotoxicity. With the increase of antibiotic resistance, the use of aminoglycosides is inevitable. Low-molecular-weight chitosan (LMWC) has shown renal protective effects in dialysis patients. However, no study has evaluated LMWC for preventing aminoglycoside-induced nephrotoxicity or determined the mechanisms underlying the renal protective effects. In this study, LMWC (165 or 825 mg/kg/day) or metformin (100 mg/kg/day) was orally administered for 13 days to rats with nephropathy induced by gentamicin (GM), a kind of aminoglycoside (150 mg/kg/day i.p. for 6 days). Both LMCW doses improved renal function. Serum creatinine levels improved in rats treated with 165 and 825 mg/kg/day LMWC (from 2.14 ± 0.74 mg/dL to 1.26 ± 0.46 mg/dL and 0.69 ± 0.12 mg/dL, resp., *P* < 0.05). Blood urea nitrogen levels were also improved in these rats (from 73.73 ± 21.13 mg/dL to 58.70 ± 22.71 mg/dL and 28.82 ± 3.84 mg/dL, resp., *P* < 0.05). Additionally, renal tissue morphology improved after LMWC treatment, and accumulation of renal methylglyoxal, a damage factor associated with carbonyl stress, was reversed. These results show that LMWC prevents GM-induced renal toxicity via a carbonyl stress-dependent pathway.

## 1. Introduction

Gentamicin (GM) is an aminoglycoside antibiotic extensively used in the treatment of Gram-negative bacterial infections. However, GM induces nephrotoxicity at low therapeutic doses [1]. Furthermore, the incidence of aminoglycoside-induced nephrotoxicity has progressively increased since its introduction, occurring in 10–25% of therapeutic courses, despite rigorous monitoring [2]. GM-associated nephrotoxicity is considered a tubulopathy-inducing renal insufficiency mediated by tubular damage and dysfunction. Aminoglycosides are freely filtered across the glomerulus. However, they are partially taken up by and concentrated in the proximal tubular cells where they cause damage. After administration, approximately 5–10% of the parenteral GM dose is retained in the renal cortex, where concentrations can exceed the concomitant serum concentration [3]. GM-induced acute kidney injury typically manifests after 5–7 days of administration. Many compounds are used to prevent GM-induced acute kidney injury, including vitamin E,* N*-acetyl cysteine, and silymarin [4, 5]. Importantly, GM nephropathy is thought to be mediated via oxidative stress [6] and cytochrome c release from mitochondria [7]; however, carbonyl stress may also be involved [8].

Carbonyl stress is produced by the binding of methylglyoxal (MGO) and its products to proteins. MGO has a 3-carbon structure with 2 carbonyl groups. Thus, it is highly active and nonselective and can rapidly and nonenzymatically react with proteins. MGO-mediated protein modification can interfere with biological activity and produce unstable advanced glycation end-products (AGEs), including* N* ^*ε*^-(carboxyethyl)lysine (CEL) [9, 10]. CEL can bind to the RAGE receptor, thereby inducing inflammation and oxidative stress [11, 12]. This type of carbonyl stress was first identified in diabetes and is regarded as the most important factor in diabetic complications [13]. Recently, carbonyl stress has attracted increased attention because of its involvement in many diseases, including nephropathy, arthritis, vasculopathy [8, 14, 15], and aging [16].

Low-molecular-weight chitosan (LMWC; 35 kDa) is more soluble, biocompatible, and biologically active than chitosan (500–1000 kDa). LMWC is used as an antibacterial, antifungal, antidiabetic, and lipid-lowering agent [17–20]. Previous studies have demonstrated that LMWC prevents type II diabetes progression in mice [21]. Furthermore, LMWC also displayed renoprotective effects in a clinical trial [22] and in experimental glycerol-induced nephropathy [23]. However, the underlying molecular mechanism of this effect remains unclear. Additionally, the effects of LMWC on GM-induced nephropathy (GN) are unknown.

Metformin is the only effective MGO-lowering agent approved by the United States Food and Drug Administration. Metformin is an antidiabetic drug that has been used for approximately 70 years. Clinical trials have shown that, in addition to controlling blood sugar [24], metformin can prevent GN [7]. Therefore, we used metformin as an MGO inhibitor to compare the renoprotective effects of LMWC.

The aim of this study was to evaluate the ability of LMWC to protect against GM-induced nephrotoxicity and to elucidate the mechanisms underlying the protective effect.

## 2. Materials and Methods

### 2.1. Chemicals

LMWC (35 kDa) was obtained from Shin Era Technology Co. (Taipei, Taiwan). GM was purchased from Yungshin Pharm Ind. Co. (Taichung, Taiwan). A BCA protein assay kit was purchased from Thermo Scientific (Rockford, IL, USA). Acetonitrile (ACN) was acquired from Merck (Darmstadt, Germany). A microalbumin kit was purchased from Good Biotech (Taichung, Taiwan). A CEL kit was obtained from Cell Biolabs Inc. (San Diego, CA, USA). Bovine serum albumin (BSA), 10% formaldehyde, MGO, citric acid, ammonium chloride, 5,6-diamino-2,4-dihydroxy-pyrimidine (DDP), and xylene were purchased from Sigma-Aldrich Inc. (St. Louis, MO, USA).

### 2.2. Animal Treatment and Sample Preparation

Seven-week-old male Wistar rats were purchased from the National Laboratory Animal Breeding and Research Center (Taipei, Taiwan). After 1 week of acclimation, the rats were divided into 5 groups with 6 rats each (the control group contained 5 rats). The control group (Group C) was injected with 0.1 mL of normal saline (i.p.) for 6 days (day 1 to day 6). The disease group (Group G) was injected with 150 mg/kg/day GM (i.p.) for 6 days (day 1 to day 6). The rats in the treatment groups were injected with 150 mg/kg/day GM (i.p.) for 6 days (day 1 to day 6), followed by administration of 165 mg/kg/day LMWC (Group Chil-G), 825 mg/kg/day LMWC (Group Chih-G), or 100 mg/kg/day metformin (Group met-G) for 13 days (day 1 to day 13). We have performed a similar experiment previously using mice and found the effective dosage to be 250 mg/kg/day. Using the following equation, we calculated the possible effective dosage for rats, which is 825 mg/kg/day. The 1/5-fold dosage (165 mg/kg/day) was also used to test whether the effect of LMWC is dose-dependent. Consider

(1) DoseRatmg/Kg  =DoseMouse(mg/Kg)   ×CoefficientRatCoefficientMouse×Body  weightRatBody  weightMouse2/3.

### 2.3. Renal Function

Serum creatinine (SCr) levels were determined by high-performance liquid chromatography (HPLC) as described previously [25]. The blood urea nitrogen (BUN) concentration was determined by a urease assay according to the manufacturer's protocol [26]. Microalbumin was assessed according to the manufacturer's protocol [8].

### 2.4. Hematoxylin and Eosin Staining and Histological Examination of Renal Lesions

Rat kidneys were fixed with 4% paraformaldehyde for 2 days, dehydrated with ethanol and xylene, and embedded in paraffin. Paraffin sections (5 *μ*m) were stained with hematoxylin and eosin. Histopathology was evaluated under a microscope. The degree of tubular lesions was graded from 0 to 5 according to the protocol of Houghton et al. [27]. The extent of the lesions was graded histopathologically from 1 to 5 depending on severity (1 = minimal (<1%); 2 = slight (1–25%); 3 = moderate (26–50%); 4 = moderately severe (51–75%); and 5 = severe/high (76–100%)). The score for each group was calculated and shown as tubular necrosis atrophy and cell infiltration.

### 2.5. Determination of MGO Concentrations in the Kidneys

Renal MGO levels were determined by HPLC [28]. Kidney homogenates were added to 2 *μ*L ammonium chloride buffer (pH = 10, to provide an alkaline environment for the reaction), and the derivative reagent, 50 *μ*L of 7.5 × 10^-4 ^M DDP, was added to the derivatization reaction. The reaction mixture was incubated at 60°C for 30 min in the dark and then layered onto an ODS column for separation (Biosil, 250 mm × 4.6 mm ID; 5 *μ*m particle size; Biosil Chemical Co. Ltd., Taipei, Taiwan). The mobile phase consisted of acetonitrile: 0.01 M citric acid buffer (3 : 97, v/v). The flow rate was 0.7 mL/min, and the excitation and emission wavelengths were 330 nm and 500 nm, respectively.

### 2.6. Determination of CEL Concentrations in Kidney Samples

All kidney homogenate samples were diluted in PBS to a final total protein concentration of 10 *μ*g/mL per the kit instructions. Samples or standard solutions (100 *μ*L) were then added to a protein adsorbent plate, which was incubated overnight at 4°C. After incubation, the plate was washed with PBS twice and incubated with assay diluent buffer for 1.5 h. The plate was then washed 3 times with wash buffer and incubated with anti-CEL antibodies at room temperature for 1 h on an orbital shaker. The plate was again washed with wash buffer 3 times and incubated with the secondary horseradish peroxidase- (HRP-) conjugated antibody for 1.5 h at room temperature on an orbital shaker. Finally, the plate was washed with wash buffer 5 times and incubated with substrate solution at room temperature for 15 min on an orbital shaker. The enzyme reaction was terminated by adding stop solution to each well. The absorbance of the reaction mixture was read immediately at 450 nm.

### 2.7. Determination of LMCW-Induced MGO Inhibition* In Vitro*

Because we found MGO to have an inhibitory effect on LMWC in the rat kidney, we wanted to know whether LMWC can bind MGO* in vitro*. To simulate human physiological conditions, we incubated MGO and LMWC together in a buffer. Sodium phosphate buffer (0.2 M, pH 7.4), which consisted of 0.2 M NaH_2_PO_4_ and 0.2 M Na_2_HPO_4_, was prepared and added to the MGO or MGO-LMWC mixture. After incubation at 37°C for 24 h, the MGO level was determined by HPLC, as described in Section 2.5.

### 2.8. Statistical Analysis

All data are expressed as the mean ± standard error of the mean (SEM) where *n* = 5-6 (*in vivo*) or *n* = 3 (*in vitro*). Differences were analyzed using one-way analysis of variance (ANOVA), and the level of significance among the various treatments was determined using the LSD multiple range test. Differences with *P* < 0.05 were considered statistically significant.

## 3. Results

### 3.1. Effects of LMWC on Renal Function

**Figure 1:**
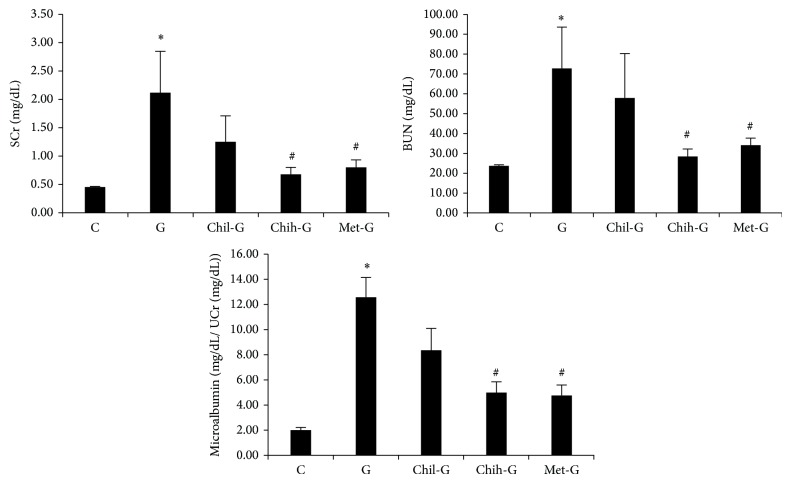
*In vivo* effects of LMWC on renal function in rats. Group C was the control group. Group G was treated with GM (150 mg/kg/day) for 6 days. Group Chil-G and Group Chih-G were treated with 165 and 825 mg/kg/day, respectively. Group met-G was treated with metformin. Serum creatinine, blood urea nitrogen, and microalbumin were altered in GN rats; however, these effects were reversed by LMWC. ^∗^ *P* < 0.05, compared to Group C; ^#^ *P* < 0.05, compared to Group G. *n* = 5-6.

Figure 1 shows the changes in renal function after LMWC treatment in GN rats. Markers of renal function, including SCr, BUN, and microalbumin, were markedly worse in Group G (SCr: 2.14 ± 0.74 mg/dL, BUN: 73.73 ± 21.13 mg/dL, and microalbumin: 12.66 ± 1.58 mg/dL), as compared to Group C (SCr: 0.46 ± 0.01 mg/dL, BUN: 24.06 ± 0.55 mg/dL, and microalbumin: 2.02 ± 0.22 mg/dL).

SCr improved in rats treated with 165 and 825 mg/kg/day LMWC (from 2.14 ± 0.74 mg/dL to 1.26 ± 0.46 mg/dL and 0.69 ± 0.12 mg/dL, resp., Group Chih-G, *P* < 0.05). BUN also improved in these rats (from 73.73 ± 21.13 mg/dL to 58.70 ± 22.71 mg/dL and 28.82 ± 3.84 mg/dL, resp., Group Chih-G, *P* < 0.05). Microalbumin also improved significantly in these rats (from 12.66 ± 1.58 mg/dL to 8.41 ± 0.76 mg/dL and 5.03 ± 0.86 mg/dL, resp., Group Chih-G, *P* < 0.05).

Treatment with LMWC significantly improved renal function in the Chih-G rats. Furthermore, treatment with metformin also improved these indicators significantly (SCr: 0.81 ± 0.13 mg/dL, BUN: 34.60 ± 3.61 mg/dL, and microalbumin: 4.80 ± 0.83 mg/dL). Therefore, treatment with LMWC for 13 days improved renal function similarly to metformin.

### 3.2. Histochemical Staining

**Figure 2:**
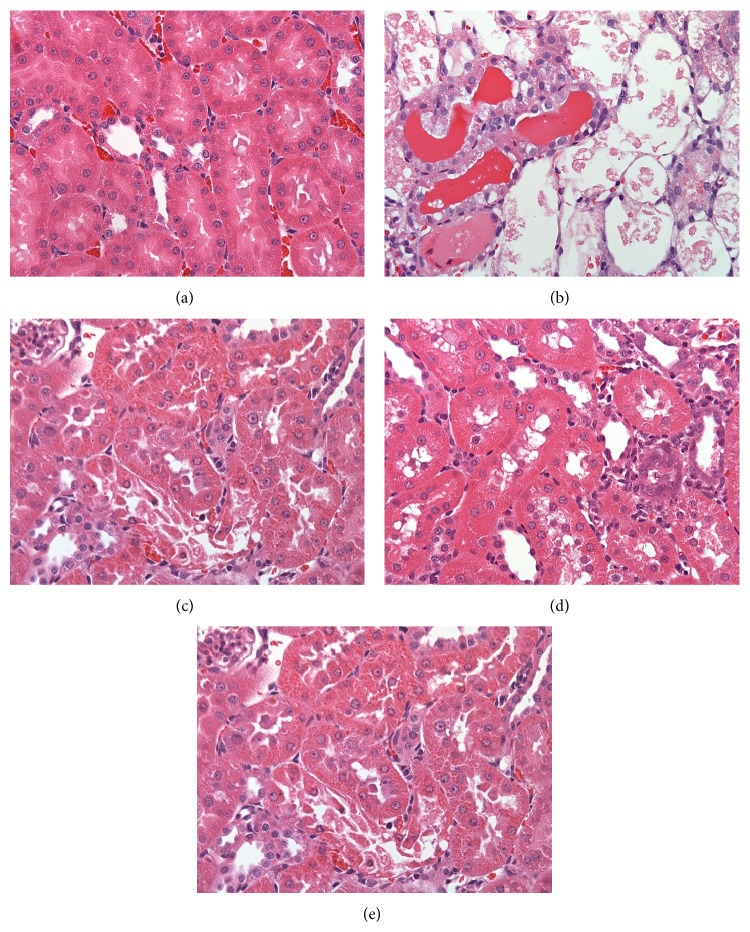
LMWC-induced changes in histology. Light micrographs of rat kidney sections were stained with hematoxylin and eosin. (a) Histology of kidney tissue in the control group. (b) Necrotic tubules and desquamation were apparent after treatment with 150 mg/kg/day GM for 6 days. (c) Treatment of GN rats with 165 mg/kg/day LMWC for 13 days improved histology. (d) Treatment of GN rats with 825 mg/kg/day LMWC for 13 days significantly improved histology. (e) Treatment of GN rats with 100 mg/kg/day metformin for 13 days significantly improved histology.

Stained renal tissue samples are shown in Figure 2. Compared with Group C tissue samples, Group G renal tissue samples showed severe tubular damage, tubular dilation, and infiltration of inflammatory cells (Figure 2(b)). Both LMWC and metformin treatment decreased the severity of tubulointerstitial nephritis and improved renal morphology (Figures 2(c), 2(d), and 2(e)) in GN rats.

### 3.3. Changes in Tubulointerstitial Histological Scores

**Figure 3:**
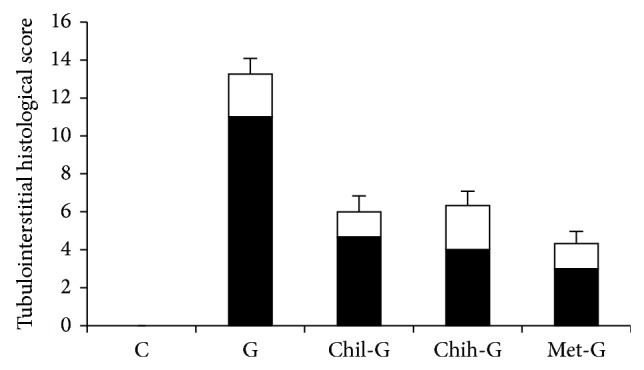
Quantitative analysis of renal tissue lesions. The tubulointerstitial histological scores were determined for tubular necrosis atrophy (black bar) and cell infiltration (white bar). Group C was the control group. Group G was treated with GM (150 mg/kg/day) for 6 days. Groups Chil-G and Chih-G were GN rats treated with 165 and 825 LMWC mg/kg/day, respectively. Group met-G consisted of metformin-treated GN rats.

As shown in Figure 3, kidney samples were examined for characteristic histological and morphological changes. Tubular necrosis atrophy was defined as tubular epithelial alterations (basophilia, degeneration and necrosis, and cell desquamation), hyaline casts, granular casts, and hyaline droplet accumulation. Cell infiltration was defined as basophilia in the tubule and inflammatory cell infiltration in the cortical interstitium and perivascular areas. Group G had the highest tubular atrophy score. Both doses of LMWC and metformin decreased the tubular atrophy score; however, only the low dose of LMWC and metformin reduced cell infiltration.

### 3.4. Effects of LMWC on Kidney MGO Levels

**Figure 4:**
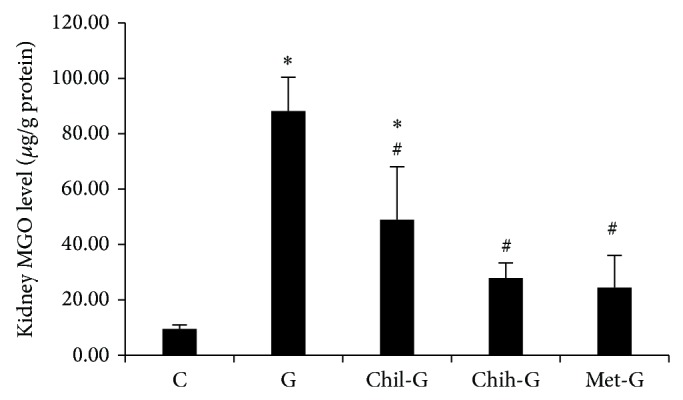
Effect of LMWC on renal MGO concentrations. MGO levels in GN rats were increased. These changes were alleviated by LMWC treatment. Group C was the control group. Group G was treated with GM (150 mg/kg/day) for 6 days. Groups Chil-G and Chih-G were GN rats treated with 165 and 825 mg/kg/day, respectively. Group met-G consisted of metformin-treated GN rats. ^∗^ *P* < 0.05, compared to Group C; ^#^ *P* < 0.05, compared to Group G. *n* = 5-6.

MGO levels were determined and normalized using a protein assay. Kidney MGO levels in Group G rats were significantly higher than those in Group C rats (*P* < 0.05, Figure 4). Treatment with LMWC (Group Chih-G) decreased MGO levels significantly (*P* < 0.05 versus Group G; Figure 4).

### 3.5. Effects of LMWC on Kidney CEL Levels

**Figure 5:**
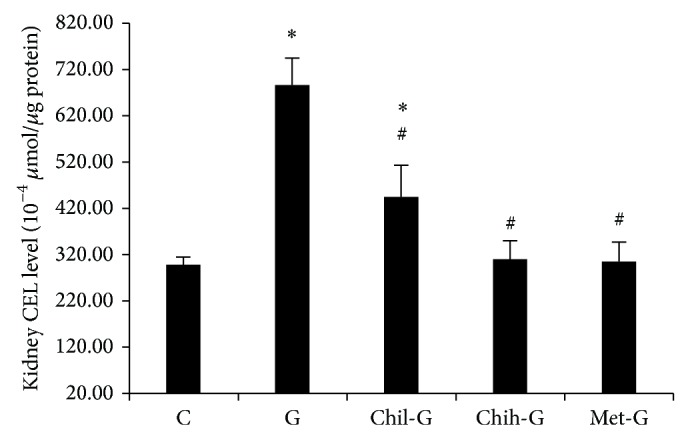
Effect of LMWC on renal CEL concentrations. CEL levels in GN rats were increased. These changes were alleviated by LMWC treatment. Group C was the control group. Group G was treated with GM (150 mg/kg/day) for 6 days. Groups Chil-G and Chih-G were GN rats treated with 165 and 825 mg/kg/day LMWC, respectively. Group met-G consisted of metformin-treated GN rats. ^∗^ *P* < 0.05, compared to Group C; ^#^ *P* < 0.05, compared to Group G. *n* = 5-6.

Rat kidney CEL levels are shown in Figure 5. CEL levels in Group G rats were twice as high as those in Group C rats. After LMWC treatment, the increasing CEL levels in the kidney lowered, as indicated by measurements in Group Chih-G rats (*P* < 0.05 versus Group G). Thus, LMWC treatment decreased CEL levels in GN rats.

### 3.6. Comparison of LMWC- and Metformin-Induced MGO Inhibition* In Vitro*

**Figure 6:**
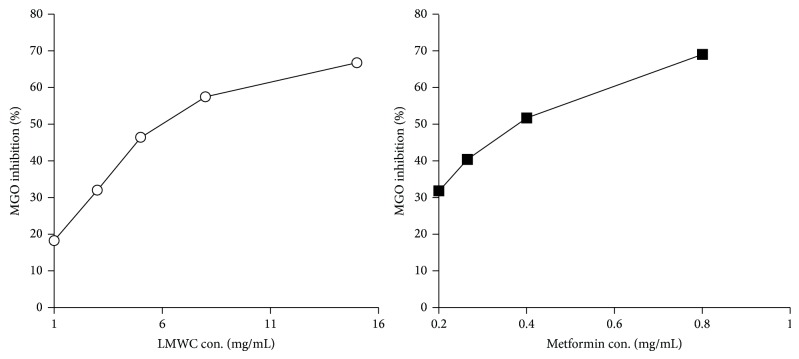
Inhibitory effect of LMWC on MGO* in vitro*. LMWC binds to MGO and decreases MGO levels* in vitro*. The inhibitory effect of LMWC on MGO binding is shown. The white circles represent LMWC and the black squares represent metformin; *n* = 3.

The inhibitory effects of LMWC on MGO activity* in vitro* are shown in Figure 6. The 50% inhibitory concentrations (IC_50_) of LMWC and metformin were 5.99 ± 0.56 mg/mL and 0.38 ± 0.02 mg/mL, respectively.

## 4. Discussion

In this study, we investigated the protective effects of LMWC in GM-induced renal injury. We found that GN rats treated with LMWC displayed improved renal function and morphology, with reduced occurrence of acute renal failure. LMWC treatment also significantly decreased renal MGO accumulation and CEL levels. Thus, our data demonstrated that LMWC decreased GM-induced nephrotoxicity by reducing kidney MGO and CEL levels.

The effects of chitosan treatment on renal failure have been investigated. When patients undergoing dialysis were treated with LMWC for 4 weeks, their renal function significantly improved relative to patients that did not receive supplements [22]. Additionally, sulfated forms of chitosan protected against renal morphological and functional changes in glycerol-induced acute renal failure [23]. Our data revealed that LMWC treatment also protects against kidney damage in the acute GN model. Taken together, our findings suggest that LMWC protects against serious kidney injury.

Many MGO-related diseases have been described [14, 15]. Several pharmacological interventions for the prevention of MGO-related injuries have been proposed, including chemical agents such as thiamine, aminoguanidine,* N*-phenacylthiazolium bromide, pyridoxamine, and metformin [29–32]. However, with the exception of metformin, most of these agents cannot be used clinically because of their toxicity, instability, and low potency. Additionally, metformin administration is not recommended in patients with renal insufficiency because of lethal adverse reactions, such as lactic acidosis. Thus, a new safe and effective agent that reduces MGO is needed. LMWC has been used widely in the pharmaceutical industry and was investigated in human clinical trials as a lipid-lowering agent [33] and antidiabetic drug [34]. Therefore, it is an ideal candidate for preventing MGO-related complications in human studies.

Metformin was used as the positive control to compare the effects of LMWC. Metformin is an antidiabetic drug used in several applications, including blood glucose regulation, renal tubular cell protection via antioxidant activity, and protection against diabetic nephropathy through preservation of podocytes. In addition, metformin is a well-known MGO inhibitor that prevents GM-induced nephropathy [7]. LMWC was renoprotective and reduced MGO to a similar extent as metformin and may thus be a promising new treatment to protect against GM-induced nephrotoxicity.

The major downstream effector of MGO is CEL [35], which is associated with diabetic complications [36] and can be formed in food via the Maillard reaction [37]. The reduction of diet-derived CEL is of great interest [38]. Antioxidants, such as *α*-tocopherol and rutin, cannot inhibit CEL formation; however, thiamin analogues, which are MGO inhibitors, can. Nevertheless, few compounds with CEL inhibitory activity have been identified* in vivo.*

In this study, we show for the first time that LMWC inhibits MGO and CEL. However, LMWC is less efficacious than metformin* in vitro*. It is worth noting that the renoprotective effects of LMWC* in vivo* are similar to those of metformin, possibly because the specific distribution of LMWC to the kidneys [39, 40] increases renal concentrations to promote the protective effect.

## 5. Conclusions

In conclusion, we show that LMWC decreases MGO accumulation and CEL levels, thereby mediating the protective effect of chitosan in GN. Given the many diseases associated with MGO and CEL, our findings may have broad applications. Nevertheless, further evidence is needed to confirm the present findings.
