# Corrigendum to “Chitosan Prevents Gentamicin-Induced Nephrotoxicity via a Carbonyl Stress-Dependent Pathway”

**DOI:** 10.1155/2017/7686249

**Published:** 2017-09-24

**Authors:** Chu-Kuang Chou, Yi-Chieh Li, Shih-Ming Chen, Yi-Min Shih, Jen-Ai Lee

**Affiliations:** ^1^Department of Internal Medicine, Chia-Yi Christian Hospital, 539 Jhongsiao Road, Chiayi City 60002, Taiwan; ^2^Department of Internal Medicine, National Taiwan University Hospital, No. 7, Chung-Shan South Road, Taipei City 10002, Taiwan; ^3^School of Pharmacy, College of Pharmacy, Taipei Medical University, 250 Wuxing Street, Taipei City 11031, Taiwan

 In the article titled “Chitosan Prevents Gentamicin-Induced Nephrotoxicity via a Carbonyl Stress-Dependent Pathway” [1], the name of the first author was given incorrectly as Chu-Kung Chou. The author's name should have been written as Chu-Kuang Chou. The revised authors' list is shown above.

Also, there was an error in Figure 2. Figures 2(b) and 2(e) were inadvertently reused from Yi-Chieh Li, Yi-Min Shih, and Jen-Ai Lee, “Gentamicin caused renal injury deeply related to methylglyoxal and Nɛ-(carboxyethyl)lysine (CEL),” Toxicology Letters, Volume 219, Issue 1, https://www.doi.org/10.1016/j.toxlet.2013.01.024. Additionally, the same picture of Figure 2(e) was presented as Figure 2(c). The corrected Figure 2 is as follows.

## Figures and Tables

**Figure 2:**
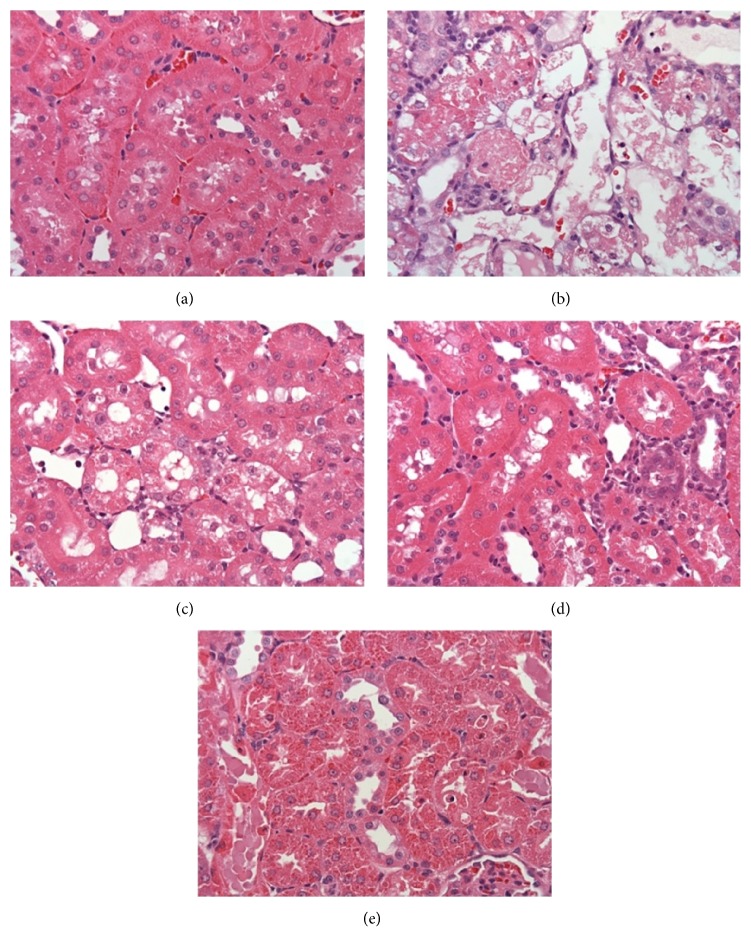
LMWC-induced changes in histology. Light micrographs of rat kidney sections were stained with hematoxylin and eosin. (a) Histology of kidney tissue in the control group. (b) Necrotic tubules and desquamation were apparent after treatment with 150 mg/kg/day GM for 6 days. (c) Treatment of GN rats with 165 mg/kg/day LMWC for 13 days improved histology. (d) Treatment of GN rats with 825 mg/kg/day LMWC for 13 days significantly improved histology. (e) Treatment of GN rats with 100 mg/kg/day metformin for 13 days significantly improved histology.
